# First Nation and Métis Needs Assessment for Pre-Surgical Rehabilitation and Education of Hip and Knee Total Joint Replacements in Saskatchewan: Uplifting Indigenous Knowledges

**DOI:** 10.1177/11786329261488704

**Published:** 2026-09-09

**Authors:** Rebecca Sawatsky, Mervin Bouvier, Eric Honetschlager, Brenna Bath, Stacey Lovo

**Affiliations:** 1 School of Rehabilitation Science, 12371 University of Saskatchewan, Saskatoon, SK, Canada; 2 Virtual Health Hub, Saskatoon, SK, Canada

**Keywords:** osteoarthritis, total joint replacement, pre-surgical, Indigenous Peoples, healthcare education, access to healthcare, rehabilitation, physiotherapy

## Abstract

**Background:**

Arthritis impacts approximately one in five Canadians, with hip and knee osteoarthritis being the most prevalent and can require total joint replacement (TJR) surgeries. In Saskatchewan, patients access pre-surgical assessments for hip and knee replacements at urban multidisciplinary clinics, and rural and remote residents face significant barriers to accessing these services. Indigenous Peoples experience disproportionally higher rates of arthritis and additional barriers accessing appropriate care.

**Objectives:**

The objective of this project was to identify the unique needs, strengths, and preferences of First Nation and Métis Peoples in accessing and receiving care for TJR.

**Design:**

An explorative qualitative approach.

**Methods:**

This study involved semi-structured qualitative interviews with 7 First Nation and Métis community members who have had or are awaiting hip or knee TJR and 6 orthopedic health care providers who have experience working with First Nation and Métis patients undergoing TJR. Health care providers included men and women, Indigenous and non-Indigenous participants. Community member participants completed a demographic and health questionnaire prior to the interview, with descriptive statistics used to determine participant characteristics. Ages of First Nation and Métis community members ranged from 43 to 75 years old (*M* = 59.57, *SD* = 10.66). Iterative thematic analysis and consensus building strategies were used to analyze data using NVivo 14 software.

**Results:**

First Nation and Métis patient perspectives highlighted five major themes: 1) Difficulty Accessing Care, 2) Community and Family Support, 3) Pre-Surgical Experience, 4) Quality of Life, and 5) Virtual Care. Health care provider interviews indicated three major themes: 1) Challenges Accessing Care, 2) Education Associated with Total Joint Replacements, and 3) Future Resource and Implementation Needs.

**Conclusion:**

The results indicated a need for increased access to culturally responsive pre-surgical education and informed recommendations for future pre-surgical rehabilitation care interventions.

## Introduction

The World Health Organization identifies musculoskeletal conditions as the leading cause of disability worldwide.^1^ In Canada, musculoskeletal conditions affected 27.8% of the population in 2017,^2^ with arthritis as the most prevalent chronic condition, affecting one in five Canadians (6 million).^3^ Projections indicate this will rise to 24% (9 million) by 2040.^4^ Osteoarthritis (OA) is a joint disease primarily affecting the knees, hips, hands, and feet.^5^ OA causes pain, stiffness, joint disfunction, and other physiological changes, restricting movement and activities of daily living.^5^ Hip and knee OA often require total joint replacement (TJR), which can relieve pain and improve function.^5^ In Canada, 69.3% of hip and 99.4% of knee replacement patients in 2021-2022 had been diagnosed with OA.^6^ Orthopedic surgeons evaluate symptoms, non-surgical management, imaging, patient expectations, physical and mental readiness, and social factors to determine appropriateness for TJR.^7^ Patients with severe pain and functional limitations are often candidates for TJR.^7^

Saskatchewan includes Treaties 2, 4, 5, 6, 8, 10, and is the Homeland of the Métis.^8^ According to the 2021 census, 187,890 Indigenous Peoples live in Saskatchewan, comprising 10.1% First Nation and 5.7% Métis.^9^ Indigenous Peoples in Canada face considerable health inequities compared to non-Indigenous populations, including disproportionally higher rates of chronic diseases such as arthritis and greater associated disability.^2,10,11^ These inequities are rooted in ongoing acts of colonization, racism, and discrimination, which continue to impact health outcomes and experiences within the healthcare system today.^12^ Such experiences have perpetuated mistrust of healthcare systems and further impede access to timely and appropriate care. In Saskatchewan, pre-surgical assessments for TJR are delivered through urban multi-disciplinary clinics that provide team-based evaluation and education.^13^ However, the centralization of these services creates barriers to access, particularly for Indigenous populations. This is reflected in the geographic distribution of Indigenous Peoples, with 43.9% living in rural or remote areas and 17.1% in small population centers.^9^ As a result, access to pre-surgical TJR care remains inequitable, highlighting a critical gap in accessible and culturally responsive services for Indigenous populations.

A systematic review indicated that education, self-management, and exercise are the top non-pharmacological recommendations for OA management.^14^ Current guidelines recommend clinicians provide education and self-management support to patients with OA, including information on self-care and disease progression.^15^ For OA patients eligible for TJR, comprehensive pre-surgical education addressing physical, psychological, and emotional aspects, and tailored to the patients cultural background and education level, can reduce anxiety and stress by improving understanding of the TJR process.^16^ Patients may benefit from educational materials in their native language to better understand TJR surgery and rehabilitation. Western healthcare may not recognize the unique needs and strengths of Indigenous cultures, and urban centered services can be inaccessible, compounding disparities due to a lack of community-informed, culturally responsive healthcare service options.

Virtual care has the potential to address long-term access disparities for residents in rural and remote areas of Saskatchewan. Research indicates that team-based and community informed virtual rehabilitation approaches enhance access to care and are experienced positively for managing musculoskeletal conditions in rural and remote communities.^17,18^ Virtual care encompasses various technologies, including video, audio, or other digital platforms.^19^ One approach uses remote presence robots (RPR), located in remote health centre centers and controlled by healthcare providers from a distant site, with integrated audio, visual, and remote navigation functions.^20^ Integrating RPR technology into community health care centres in a Northern Inuit community in Canada resulted in high patient satisfaction, with 95% of participants indicating they would use a robot again.^21^ Virtual care can facilitate access to specialized services, such as physiotherapy, thereby reducing the burdens of travel.^17,18^ This education can be tailored to meet the unique needs of Indigenous Peoples, helping patients understand what to expect, address concerns, and make informed healthcare decisions. Despite rising community requests for home-based health care delivery, the impact of virtual care on patients’ pre-surgical educational needs remain unevaluated.

First Nation and Métis perspectives on orthopedic pre-surgical assessment and education needs have not yet been investigated. Further, no known hip and knee replacement programs in Canada are designed and delivered in an Indigenous-informed, community-led manner. Research indicates that primary health care for Indigenous communities is strengthened when services are designed to meet community-specific needs or owned and managed by communities themselves.^22^ To our knowledge, no studies have explored virtual services for pre-surgical assessment and education for First Nation and Métis populations. Engaging Indigenous communities from the onset is crucial for optimal health outcomes, and incorporating virtual processes will enable remote members to access programming otherwise unavailable to them.

This project focuses on enhancing equitable access to care through a needs assessment developed and conducted in partnership with Indigenous community members to identify unique needs, strengths, and preferences of First Nation and Métis Peoples in accessing and receiving pre-surgical care for total joint replacements. The research aims to better understand specific pre-surgical needs to enhance culturally responsive access to equitable care for First Nation and Métis Peoples in Saskatchewan accessing total joint replacements.

## Methods

This project follows a wâhkôhtowin and Ethical Space framework.^23-25^ Wâhkôhtowin is a Cree and Métis teaching of kinship and relational responsibility, emphasizing community and the importance of building, honoring, and maintaining good relationships.^23,24^ While specific to Cree and Métis teachings, and may not be applicable to all Indigenous worldviews, its core relational principles helped guide this project. To honour First Nation and Métis patient perspectives, a patient-oriented approach was utilized through collaboration with two patient partners (MB, Métis Elder) and (EH, First Nation). In patient-oriented research, patients engage as partners, contributing lived experience to ensure that project outcomes will be meaningful to patients.^26,27^ The two patient partners involved in this project (MB, EH) collaborated with the research team on analysis, manuscript development, and knowledge translation. The patient partners on this project were invaluable in ensuring that the recommendations and results were meaningful to patients. Ethical space is a theoretical framework that occurs when two distinct societies, with differing worldviews and cultural backgrounds, engage with each other, creating a space that acknowledges differences while fostering collaboration between Indigenous and Western cultures.^25^ This project aims to create an ethical space by bridging Western health care provider perspectives with lived experiences from First Nation and Métis patients.

When conducting research in First Nations communities, it is essential to respect and uphold the OCAP principles: Ownership, Control, Access, and Possession, which ensures that Indigenous participants retain control over the research process.^28^ Métis Data Governance Principles guided work with Métis participants.^29^ This project involved Indigenous community members from across Saskatchewan, and not one specific community, therefore, principles were adapted to respect each participant’s needs and rights. This included conversations about comfort, interview format, anonymity, and the option to review transcripts. The transcript review process included one week where participants could add, edit, or remove content from the transcript to ensure data collected was accurate of their experience. Participants had the option to use a translator if needed. First Nation and Métis patient participants received an honorarium of $50 for community members and $100 for Elders and Knowledge Keepers in appreciation of their time.

We recognize that our identities, experiences, relationships, and positions shape how we approach this research and our relationships with communities and patient partners. We therefore provide the following positionality statements to acknowledge the perspectives and responsibilities we bring to this work. RS is a woman, master’s student, and fourth-generation white settler in Canada. She has worked in partnership with First Nation and Métis communities as a research assistant for five years and has developed relationships with the patient partners in this project for two years. SL is a woman and fourth-generation white settler in Canada. She has worked in partnership with First Nation and Métis communities in Saskatchewan for 13 years in collaborative research projects on access to musculoskeletal healthcare and in partnership with the patient partners for two years. BB is a woman and fourth-generation white settler who has worked in partnership with First Nation communities in Saskatchewan for eight years on collaborative musculoskeletal health projects. RS, SL, and BB live and work on Treaty 6 Territory and the Homeland of the Métis and are grateful for the opportunities that Indigenous lands provide them and their families. MB is a Métis man and Elder, living in Île-à-la-Crosse, and has a long history of advocacy for Métis health in Saskatchewan. EH is a Cree man and teacher from Muskeg Lake First Nation with a passion for education.

Recruitment of participants was facilitated through the University of Saskatchewan’s School of Rehabilitation Science and Saskatchewan Network Environment in Indigenous Health Research (SK-NEIHR) Facebook pages, Saskatchewan Health Authority, community health facilities, and private physiotherapy clinics. Recruitment information was posted on the previously listed social media sites or displayed on the walls in the waiting rooms with research team contact information in email and phone number format. Snowball sampling was also utilized as a recruitment strategy. Interview guides were co-created by the research team (Appendix 1 and Appendix 2). Inclusion criteria were: 1) First Nation or Métis adults (self-identified) over 18 years old who have had or were on the waitlist for TJR in Saskatchewan or 2) health care providers who had experience directly working with Indigenous patients receiving TJR in a pre- or post-surgical capacity in Saskatchewan. Volunteers were excluded if they were non-Indigenous, had undergone TJR outside Saskatchewan, or were not actively on the surgical waitlist. Health care provider participants were excluded if they were not directly involved in TJR care or were employed outside of Saskatchewan. Participants were contacted by email or phone and provided study information and consent forms by a member of the research team (JM, RS, SL). Interviews were scheduled with adequate time for consent review and questions. At the start of the interview, a research team member (RS) reviewed the project in detail, the reason for research on access to care for TJR with First Nation and Métis Peoples, and consent. Participants provided oral or written consent. Relationship building began at this time with patient participants through mutual discussion and were maintained throughout the interview process and research project. This study was approved by the University of Saskatchewan Behavioural Research Ethics Board (Beh ID #2665) on May 25, 2021. Interviews with health care providers were completed by a Master of Physical Therapy student (JM) at the University of Saskatchewan and began in July 2021 and commenced August 2021. Interviews with First Nation and Métis patients were conducted by RS and began in August 2022 and commenced June 2025.

An exploratory design employed semi-structured interviews with First Nation and Métis patients who had received surgery or were on a TJR waitlist in the province (Appendix 1) and with health care providers experienced working with First Nation and Métis patients undergoing TJR in Saskatchewan (Appendix 2). This method allowed in-depth exploration of patient and provider experiences, with flexibility for follow-up questions based on responses. Interview guides were developed by the research team. Interviews were completed virtually, through Zoom, phone, or remote presence robot (RPR) technology with a member of the research team. Interviewers (RS, JM) were trained by the same researcher (SL). Audio recordings were transcribed using Otter AI and reviewed and corrected by the research team (RS).^30^ Otter AI is an AI-assisted transcription software that is hosted on servers in the United States, utilizing AWS S3 storage and enables AWS SSE on data. Prior to interviews, First Nation and Métis patients completed a health and demographic questionnaire (Appendix 3) with the research team. The purpose of the questionnaire was to obtain health and participant data.

Quantitative data were analyzed using IBM Statistics Software Package (SPSS: Version 31) to determine descriptive statistics of participant characteristics.^31^ Qualitative data were analyzed using an inductive coding process with the software program NVivo 14.^32^ Iterative thematic analysis and consensus building strategies were used to build themes from patient and health care provider experiences.^33,34^ This is a qualitative data analysis method where themes are continually developed and refined throughout the research process.^35^ RS initially developed codes, which were then reviewed with SL and final codes were determined through consensus. RS identified themes within the data as a first-level analysis. A secondary-level analysis was completed by SL. RS individually met with SL and patient partners MB and EH to refine themes. Final themes and subthemes as well as future recommendations were determined through collaboration with patient partners (MB, EH). This process involved knowledge sharing on behalf of the First Nation and Métis patient partners to ensure prioritization of Indigenous knowledges. A reflexive approach was integrated throughout the research, with team members continually reflecting on how their experiences and biases could influence study outcomes.

Qualitative rigor was assured through a consistent analysis process determined with RS and SL, regular meetings throughout analysis by the research team and patient partners to ensure consistency in process. Additional measures to include qualitative rigor included member checking with patient partners, peer debriefing between RS and SL, careful use of codebooks, and ongoing reflexivity. RS utilized notes and annotations to track her own process through analysis. Sample size was guided by the concept of data saturation, in which no new themes or insights emerged within the last few interviews in both patient and HCPs.^36^ Sample size was further informed by the concept of information power, where the more relevant and rich information participants hold in relation to the study aim, the fewer participants are required. Given the focused study aim, use of semi-structured interviews, and inclusion of participants with direct lived experience, the sample of seven First Nation and Métis patients and six HCPs was sufficient to generate rich, in-depth data.^37^ Relationship building was the foundation of this process, where RS engaged in ongoing conversations with MB regarding culturally responsive care and challenges Indigenous patients face in navigating the provinces healthcare system, informing recommendations and future research.

## Results

The demographic questionnaire was completed by seven patient participants (Appendix 3). Ages ranged from 43 to 75 years old (*M* = 59.57, *SD* = 10.66). 57.1% of participants were women, and 42.9% men. All of these participants had previously had a TJR. Six (85.7%) who had undergone a total hip, with one (14.3%) who had received a total knee replacement. One participant (14.3%) had also been on the waitlist to receive a knee replacement. Duration of pain experienced ranged from six to 30 years (*M* = 17, *SD* = 10.42). All participants indicated that their pain limited their ability to participate in cultural practices. Participants responded to open-ended questions about cultural activities and traditional healing methods with a general reduction in participation and various methods used for pain management. Participant characteristics are included in Table 1. Qualitative responses to open-ended questions surrounding culture are included in Table 2.

**Table 1.** Participant Characteristics (N = 7)

Variable	Mean ± SD	Range (min – Max)
Age (years)	59.57 ± 10.66	43-75
Pain Duration (years)	17.00 ± 10.42	6-30
Waitlist Duration (months)	2.0 (single value)	2-2

Variable	Category	N (%)
Gender	Woman	4 (57.1)
	Man	3 (42.9)
	Other/Not Reported	0 (0)
Sex Assigned at Birth	Female	4 (57.1)
	Male	3 (42.9)
	Prefer not to answer	0 (0)
Osteoarthritis Diagnosis	Yes	2 (28.6)
	Other/No Diagnosis	5 (71.4)
Post-Surgical	Yes	7 (100)
	No	0 (0)
Post-Surgical Joint Replaced	Hip	6 (85.7)
	Knee	1 (14.3)
	Other	0 (0)
Currently on Waitlist	Yes	1 (14.3)
	No	6 (85.7)

**Table 2.** Qualitative Findings From Demographic Questionnaire

Question asked	Response
Please describe the cultural practices that are limited by this problem.	P: Social activities.
	P: Cannot leave [the house], have difficulty walking and being social.
	P: Everything – all cultural practices.
	P: Walking was difficult. Used a walker or cane depending on pain and how long they had to walk for.
	P: Swelling and pain makes participation difficult.
	P: Difficult to go to a smudge, cannot do any round dances, cannot bounce, etc.
	P: Since the surgery no real limitations. Prior to the surgery even hunting and fishing were often painful, especially hunting.
Are there any traditional methods you use for your hip and/or knee problems that you would like your health teams to understand?	P: Understanding your body as a person. The individual is more efficient.
	P: Muskrat root as a pain killer. Can make this as as tea or eat it.
	P: No.
	P: Smudging on right knee specifically.
	P: Pain salve they made.
	P: No.
	P: The occasional sweat. No use of any medicinal ingredients. Not here, I don’t like taking medication any kind.

In total, there were 13 participants in the project. Seven First Nation and Métis patients were interviewed, with seven post TJR, and one participant also being on the waitlist. Interview duration varied from sixteen to forty minutes. Six HCPs were interviewed: five physiotherapists and one orthopedic surgeon. Health care providers included men and women, Indigenous and non-Indigenous participants. Interview duration ranged from eighteen to thirty-one minutes. All participants completed required data collection.

## First Nation and Métis Patient Perspectives

**Figure 1.**
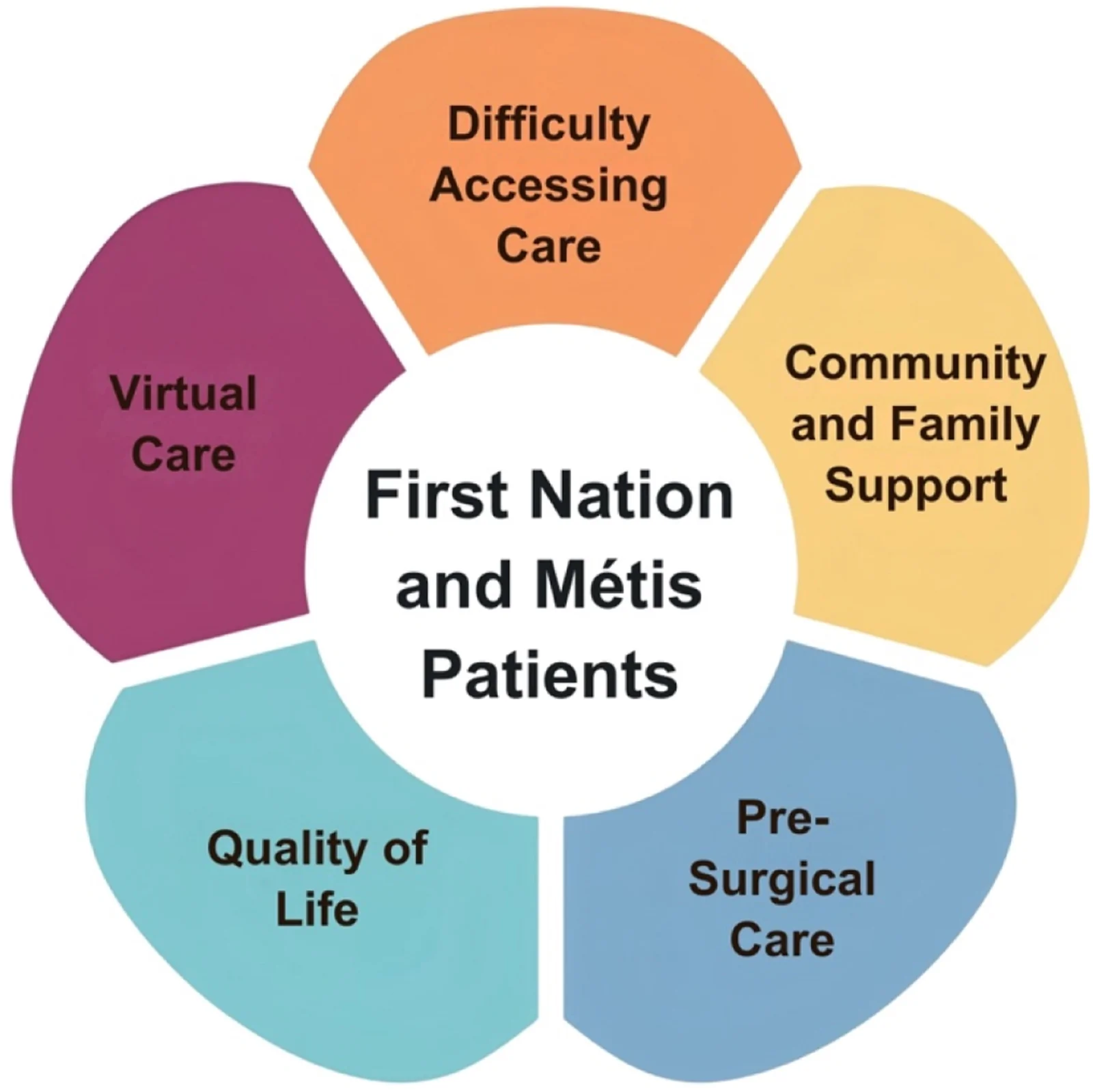
First nation and Métis patient major themes

First Nation and Métis patient perspectives included five major themes: 1) Difficulty Accessing Care, 2) Community and Family Support, 3) Pre-Surgical Experience, 4) Quality of Life, and 5) Virtual Care. Figure 1 depicts the major themes identified by First Nation and Métis patient perspectives. Additional quotes for each theme are included in Appendix 4.

### Difficulty Accessing Care

Patients described experiences that contributed to **Difficulty Accessing Care** for their TJR. Challenges included obtaining coverage for necessary equipment or services, communication breakdowns due to speaking different languages, and the need to leave their community to receive health care. Subthemes were: 1) Financial Strain of Healthcare Needs, 2) Language: A Lot of People Don’t Understand, and 3) Extensive Travel Requirements.

Patients frequently needed mobility equipment post-surgery, adding to the **Financial Strain of Healthcare Needs**. Patients expressed a desire to be informed in advance about required walking aids or other equipment, as unexpected expenses caused financial stress. One patient described the additional financial strain of attending physiotherapy, chiropractic, and other pain-management appointments before their TJR. Another noted the difficulty of taking time off work for pre-surgical appointments, which sometimes prevented attendance altogether.

Several patients noted that speaking a different **Language** made communication with health care providers more difficult. One patient noted that providers may assume patients understand information based on body language, but this may not always reflect true comprehension, particularly among individuals whose first language is not English: *“Some of those older people just don’t understand. They’ll just nod their head yes, even if they don’t know what that person’s saying. So, you think they understand, but really, they don’t have a clue and they’re just nodding yes.”* One patient, whose first language is Michif, described the disconnect they feel when communicating in English: *“Your first language, you understand your identify, and then a lot of people don’t understand that identity.”* Several patients described a desire for pre- or post-surgical educational materials translated into their own languages. Some noted that a video format would be more suitable than written materials, as they do not read or write in their language.

Although all patients in this study were able to travel to their appointments for a TJR, **Extensive Travel Requirements** were consistently reported as a significant burden due to planning involved and impact of unpredictable weather. Travel times ranged from 30 minutes to five hours one way, and many patients had to stay overnight after appointments, taking time away from their families. Multiple patients emphasized the burden of travel: *“It’s not the case of just a quick drive to the doctor right, it’s like three close to four hours”* and *“I always stay the night because I wanted to rest … because it’s tiring, you know, driving.”* One patient described driving themselves home after a hip replacement, highlighting the physical and logistical challenges involved: *“I wasn’t supposed to drive. I had a friend to drive my truck, but she was too short, and she said I can’t drive, so I had to drive.”*

### Community and Family Support

The theme, **Community and Family Support**, encompasses the need for support and healthcare within one’s home community, an increased knowledge surrounding First Nation and Métis culture within the Saskatchewan healthcare system, and the importance of family in patients’ pre- and post-surgical care. Subthemes were: 1) Community-Based Rehabilitation Needs, 2) Cultural Awareness and Understanding, and 3) Family Support: “I’m Glad I Had Somebody to Help Me”.

**Community-based Rehabilitation Needs**, particularly access to a physiotherapist within one’s home community, was continually mentioned by patients: *“I wish there would have been more available for physio close by, like being so far North up there. There was just nothing, no options.”* While one patient had a physiotherapist in their Northern community, they noted the burden on providers due to high patient volume. One participant expressed a desire for a dedicated space to exercise within their own community, stating that, *“it would be nice if there was an exercise room here”* where people could meet and exercise together in a group setting.

Participants expressed a need for greater **Cultural Awareness and Understanding** within the healthcare system. Transparency concerning cultural liaisons in hospitals was important to multiple patients, as many were unaware of their support until surgery. To improve this process, patients recommended providing a list of cultural liaisons in major health centres across Saskatchewan. Further discussion occurred around differences between Métis and First Nation cultures depending on the region and stressed the importance of understanding each patient’s beliefs and how these may impact their care: *“We have different customs in the northwest, you know, we have our language, we have our culture, and we have our cultural diversity”*.

**Family Support**: **“I’m Glad I Had Somebody to Help Me”**, was described as essential to pre- and post-surgical recovery. In some cases, this support was intensive, with one patient noting that *“[my daughter] moved in here during a couple months after every surgery.”* More broadly, patients emphasized the importance of having consistent support, with one stating, *“without my wife, I don’t think I would survive … that’s where you know that support is. And I’m very thankful that I have that.”* Others highlighted the emotional and physical benefits of proximity to family, explaining that they had *“family in both communities or close by,”* while another reflected that recovery would be particularly difficult for those without such support: *“for single people, it’s gonna be hard… we can’t get around. Like I’m glad I had somebody to help me.”*

### Pre-Surgical Experience

Patients reported mixed experiences when accessing pre-surgical care. Many described reaching out to family doctors about their pain but felt dismissed or believed the extent of their pain was misunderstood. Pre-surgical education varied, and while some had a comprehensive understanding, others experienced significant gaps. Subthemes included: 1) Appropriateness of Pre-Surgical Care, 2) Availability of Physiotherapy Services, and 3) Variable Quality of Education Provided.

Patients described significant barriers related to the **Appropriateness of Pre-Surgical Care**, including long wait times, limited continuity of care, and challenges obtaining appropriate referrals. Difficulty accessing care was common, with one patient noting that *“even for a regular appointment it’s up to six months”* to see their family physician. Others reported a lack of continuity due to not having a regular family provider, explaining that *“you never got the same doctors … there was no follow-up, like nobody seemed to know your history.”* Several patients felt that their pain was minimized by providers, contributing to delays in referral and treatment. In more severe cases, delayed recognition of symptoms led to prolonged suffering and reduced quality of life. One participant described years of having their symptoms attributed to other conditions, such as anxiety, depression, or fibromyalgia, before finally receiving a referral and eventually a bilateral hip replacement.

Patients recalled the **Availability of Physiotherapy Services** they had accessed prior to their TJR. Unless they had sought out private physiotherapy, pre-surgical physiotherapy care was not received. When asked if they had received any treatment to prepare for surgery, exercises, or services and supports patients responded that: *“No, they didn’t give me anything like that.”* While some had access to funding for necessary equipment, others relied on family support to obtain walking aids.

Finally, patients reflected on their experiences with the **Variable Quality of Education Provided** during the pre-surgical process. Many described feeling confident entering surgery with their knowledge level after receiving detailed information about the procedure, including risks, surgical steps, and recovery. Despite this, several patients indicated that they did not fully understand all aspects of their TJR. One patient stated that although information was presented in multiple ways, *“I didn’t understand it.”* Feelings of being rushed during pre-assessment also contributed to gaps in understanding, with one patient explaining that *“you really didn’t have too much time to read the papers … because I’m a slow reader,”* adding that *“they rush you. They’re so used to doing all this that they’re like, You know what? This is how it goes, hurry, hurry, hurry.”* To compensate, some patients sought additional information independently, often through personal research online. However, others proceeded to surgery with gaps. When one patient was asked if they felt prepared for surgery they replied: *“No, I don’t really know what was going on.”*

### Quality of Life

Patients described the impacts on their **Quality of Life** both before and after surgery. Patients reported reducing participation in exercise, social, and familial activities due to pain. However, numerous participants noted improvements in QOL following surgery, including enhanced physical function and mental health. Subthemes included: 1) Cultural Participation, 2) Experiencing Functional Decline, 3) Emotional Burden of Chronic Arthritis, 4) Post-Surgical Pain, and 5) Patient-Centered Rehabilitation.

The subtheme of **Cultural Participation** showcased the extent to which pain and reduced mobility limited patients’ ability to engage in cultural activities. One patient described being unable to participate in sweat ceremonies due to mobility constraints, explaining: *“I used to go into the sweats … but I can’t no more … I can go into the sweat, but I can’t get out. They’d have to carry me out, so I don’t even go anymore.”* Another described the emotional impact of reduced participation in cultural gatherings*: “I’m with the old kookums in the back on lawn chairs … I kind of feel like an imposter, honestly, like I’m not old enough to be there, and people don’t see it*.*”*

Patients **Experiencing Functional Decline** were severely impacted by pain both pre- and post-surgery. Prior to TJR, many reported giving up lifelong activities and experiencing significant functional decline. Patients described having to stop activities they once routinely engaged in, such as hockey or mowing their lawn. Others highlighted considerable limitations in basic mobility, noting that, *“before my surgery, usually I go for like a walk. I can’t even do that.”* Pain disrupted normal activities of daily living, with one patient describing repeated stops while walking and needing to rest multiple times before reaching home. Despite these challenges, many expressed significant relief as movement and function improved post-surgery: *“I’m just so happy I had it done. You know, it’s probably going to add years to my life. Yeah, do things without more pain.”* However, improvements were not universal. While several patients reported gains in mobility post TJR, others continued to experience limitations and pain: *“I’m still not what I used to be, though. So, it’s kind of depressing honestly, remembering what I was like 10 years ago compared to today”*.

Struggles with **the Emotional Burden of Chronic Arthritis** were a significant aspect of patients QOL. Patients described the emotional burden of chronic pain, including anger, frustration, and exhaustion over no longer being able to do activities that were previously managed easily. Several patients expressed fear and anxiety related to surgery itself, including concerns about complications and not waking up from the procedure: *“I was pretty scared … like at first I was afraid. Oh no, I didn’t wake up, you know, like something could happen. So, I was scared*.*”* This fear sometimes resulted in cancelling appointments, with one patient noting, *“I had so many different cancellations because I was afraid with surgery.”* However, one patient reflected on meaningful emotional improvement following surgery: *“Just agony of it, you know, and the agony of it was painful. Today I don’t have that agony. I feel happy. I feel, you know, joyful.”*

**Post-surgical Pain** impacted multiple facets of patients QOL including sleep and social activities. Some patients described difficulty sleeping due to ongoing discomfort and others relied on pharmacological management to maintain daily functioning A consistent theme was living with persistent pain while continuing to push through daily life. Several patients expressed disappointment post-surgery, as they had expected substantial or complete pain relief following their TJR. In contrast, other patients reported a significant reduction in pain following surgery, along with a return to pre-pain levels of function. One patient stated, *“after they put they changed the hip, I became a different person … after surgery, oh my, I never felt so relieved … they just changed my whole life*.*”*

Finally, patients reported mixed experiences with post-surgical **Rehabilitation** and expressed a desire for more **Patient-Centered** models of care. Some encountered challenges performing exercises independently at home and noted a lack of in-person physiotherapy options in rural and remote areas. Several patients emphasized the need for accessible support and hands-on guidance to ensure exercises were completed correctly as a future recommendation. Conversely, others described feeling confident in managing their rehabilitation exercises independently.

### Virtual Care

Patients had varying perspectives on accessing health care virtually, with some accepting it, and others preferring in-person care. Several patients described familiarity with virtual communication through work, school, and personal activities, noting that *“it’s just a part of life now.”* Others highlighted the widespread availability of cellphones, even in more remote contexts: *“Even up North fishing like everybody’s got a cell phone.”* Conversely, concerns were raised about accessibility for older adults and those less comfortable with technology. One patient observed that while their own experience with virtual care was positive, *“people older than me, I don’t know if they could handle it,”* while another similarly noted that *“some old people, they don’t even know how to turn on a phone.”* Patients also questioned the effectiveness of virtual formats for learning new skills, suggesting that *“hands on doing it they would remember … but virtual, they would have a tough time trying to. They’ll do it once, but they won’t do it again.”* Despite these concerns, some patients expressed a preference for virtual care, particularly when travel burdens were high. One patient described attending a brief in-person appointment that required significant travel, reflecting that *“I only seen him like two, three minutes. He came in, looked at me, and I walked and then he said, “We’ll do surgery”.*

## Health Care Provider Perspectives

**Figure 2.**
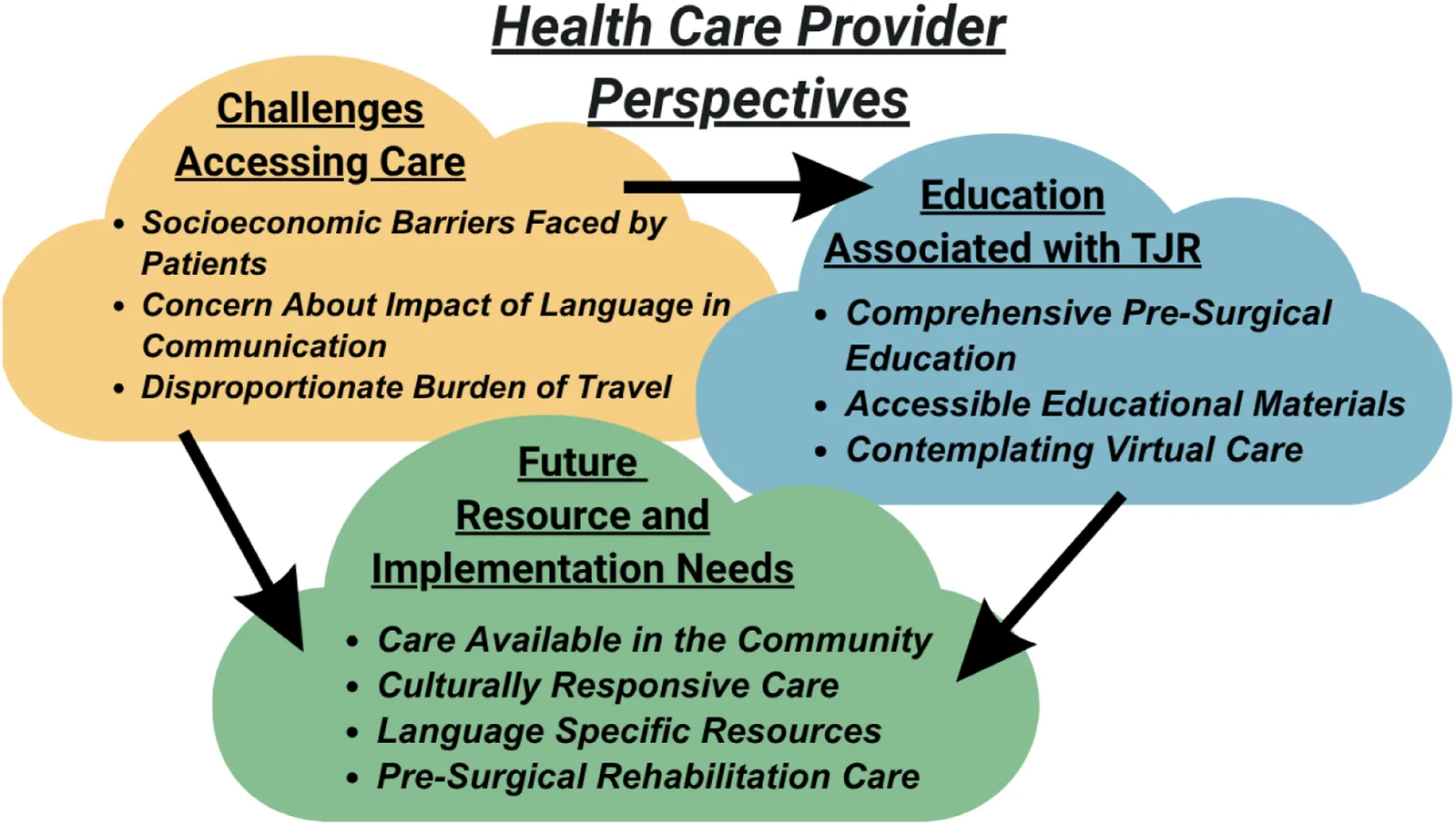
Health care provider major themes

Health care provider (HCP) perspectives from five physiotherapists and one orthopedic surgeon included three major themes: 1) Challenges Accessing Care, 2) Education Associated with Total Joint Replacements, and 3) Future Resource and Implementation Needs. Figure 2 depicts the major themes and subthemes identified by HCPs. Additional quotes for each theme are included in Appendix 5.

### Challenges Accessing Care

The theme, **Challenges Accessing Care**, encompasses the challenges HCPs face in pre- and post-surgical care that affect patient experience and quality of care. Delays in equipment funding, language barriers hindering communication, and long travel times place a burden on patients. Subthemes included: 1) Socioeconomic Barriers Faced by Patients, 2) Concern About Impact of Language in Communication, and 3) Disproportionate Burden of Travel.

Health care providers continually raised concerns about funding, and the **Socioeconomic Barriers Faced by Patients** when accessing care. In Saskatchewan, publicly funded physiotherapy services prior to TJR surgery are not available, requiring patients to access private services at their own expense. HCPs emphasized the influence of socioeconomic status on recovery outcomes, suggesting that patients with greater financial resources were better positioned to access supportive services. Patients also identified this as a critical issue; however, they provided specific examples demonstrating that this burden occurred at multiple points throughout the care pathway.

HCPs **Concern About the Impact of Language in Communication** highlight how language barriers create challenges in care delivery, making it difficult to assess patients’ understanding and optimize care. HCPs had various perspectives to share regarding treating patients with a language barrier: *“I did have one patient who mostly spoke Cree. So, there was kind of a language barrier. We were able to communicate, and we didn’t have too much trouble, but he’d always look towards his daughter who was translating for him. So, I mean, probably things got misunderstood.”* Beyond patient encounters, providers also highlighted broader implications for overall access to care, suggesting that language barriers may deter individuals from accessing treatment altogether, with one orthopedic surgeon explaining that *“some people don’t seek out care because they don’t have the means of communication.”* Patient participants agreed with this perspective and further clarified that these challenges included difficulties in understanding educational materials and clinical recommendations to support outcomes.

Finally, the **Disproportionate Burden of Travel** on patients was further identified by physiotherapists and an orthopedic surgeon as a significant barrier to accessing services and matched the impressions of the patients. In some regions, patients may need to travel long distances on gravel roads to access physiotherapy services. Transportation burdens were further compounded when patients were required to travel substantial distances for brief appointments, with HCPs noting the disproportionate burden placed on patients: *“They’re on the road for 12 hours, they were in a hotel, and they got to see the doctor for five minutes and they didn’t even really look at their knee, or their hip, or their shoulder. Just kind of looking at an x-ray and having a quick conversation.”* Given these challenges, a physiotherapist offered a potential solution that utilizes virtual care: *“A five-minute ortho visit that could just as easily be an x-ray done at the (rural community name redacted) hospital, gets sent to the ortho, and then they have a phone call or a video conference.”*

### Education Associated With Total Joint Replacements

The theme, **Education Associated with Total Joint Replacements,** involves perspectives centered around improving the education process for patients receiving a TJR. Subthemes included: 1) Comprehensive Pre-Surgical Education, 2) Accessible Educational Materials, and 3) Contemplating Virtual Care.

HCPs emphasized the need to expand **Comprehensive Pre-Surgical Education** to include information on treatment options, pre-surgery preparation, pain, and expectation management. Gaps were identified in current education, particularly regarding the nature of arthritis, pain mechanisms, and the role of exercise prior to surgery. As one provider explained, *“the most important thing is understanding what is causing their pain and dysfunction … why they feel the way that they do,”* stressing the need for improved education earlier in the care process. Providers also noted that reinforcing the importance of exercise prior to surgery could improve patient readiness. One physiotherapist highlighted the importance of managing patient expectations around pain, explaining that patients often perceive their pain as purely mechanical, when in reality it may not be fully resolved by a TJR alone. This perspective closely aligned with patient accounts, as one patient similarly expressed disappointment with ongoing pain following surgery.

Health care providers described the range of **Accessible Educational Materials** available to patients in Saskatchewan undergoing hip and knee TJR surgery. These included standardized provincial materials and exercise guides. Beyond formal educational materials, providers emphasized the benefit of social support, where patients can connect with others who have undergone similar surgeries. One orthopedic surgeon described the advantages of their educational website, which provides patients with comprehensive information. They also acknowledged challenges in determining what information to include given the varying socioeconomic backgrounds of their patients. However, they did not speak to accessibility of this information for patients who don’t have access to technology. Patients had commented on difficulty with the presentation of educational materials; however, this detail wasn’t present in HCP interviews, perhaps speaking to an area of potential improvement in communication. Assurance of understandability of educational materials for patients should be made prior to surgery moving forward.

Finally, providers had discussions **Contemplating Virtual Care** and its potential role in the pre-surgical process. While virtual care was viewed as increasingly integrated into practice, providers noted that older adults may face barriers related to digital literacy and access to technology. An orthopedic surgeon highlighted the efficiency of virtual appointments for delivering pre-surgical education, noting that much of their role involves communicating information that is well suited to a virtual format. However, physiotherapists emphasized the importance of synchronous, interactive education formats that allow for real-time discussion and questions. Despite these advantages, physiotherapists cautioned against a heavy reliance on virtual care, as personal connections with patients are crucial: *“We might be going in the wrong direction if we start going virtual. Just a lot of building relationship, that language, the talking to a person, having that contact with people is very, very important.”* Patients on the other hand, expressed more complexity with the consideration for virtual care. This included some being in favour and comfortable with virtual care due to the popularity of cell-phone use. However, patients cautioned that older adults may not be as familiar with technology. Some patients were anxious to consider virtual care due to the potential for travel savings.

### Future Resource and Implementation Needs

HCPs identified a **Future Resource and Implementation Needs** theme with four subthemes: 1) Care Available in the Community, 2) Culturally Responsive Care, 3) Language Specific Resources, and 4) Pre-Surgical Rehabilitation Care.

Several providers identified making resources readily available to support patient access to **Care Available in the Community** as a key goal for enhancing delivery, emphasizing the importance of including educational and support materials locally. Physiotherapists discussed the potential value of delivering services directly within rural and remote communities, as we also heard from patients, although they noted that this was not yet part of their current roles. One provider suggested *“to actually have me there or any physical therapist or anybody doing the actual pre-assessment clinics or pre-assessment stuff, we need somebody to go there.”*

Providers highlighted the importance of ensuring **Culturally Responsive Care** within the pre-surgical education process. Several emphasized the need for culturally relevant content within educational materials. Family involvement was identified as an important support for patients, particularly during pre-surgical education, with one provider stating, *“it’s helpful when they can have a family member, somebody in with them during their pre-op stuff.”* In addition, increasing representation of First Nation and Métis health care providers and support staff was identified as a recommendation. Despite recognition of these gaps, providers acknowledged that culturally responsive content was not currently integrated into practice: *“Honestly, we don’t do much of anything in regard to anything culturally sensitive or even awareness.”* Patients had agreed with the importance of culturally relevant educational materials as well as family support. They also reinforced the importance of understanding Indigenous culture and cultural diversity. Given that HCPs reported that culture isn’t currently integrated into care, this speaks to the importance of system support for Indigenous cultural education to ensure understanding of its importance in health outcomes and how providers can integrate these lessons into patient interactions.

Integrating **Language Specific Resources** was expressed as a potential future implementation that would benefit patients, specifically older adults. Currently, HCPs noted that family members often assist with translation when needed; however, this is not universally available, highlighting the variability of translation support for patients. Aligning with patient perspectives, several physiotherapists discussed the value of translating existing provincial resources to improve accessibility, particularly for patients with limited English proficiency. Specifically, a few commonly spoken Indigenous languages in Saskatchewan were noted as a starting point: *“revamping the entire book into Cree, let’s say, or Dene. Cree, I would say probably is valuable. I would say that would bring value to Indigenous populations for sure.*”

Finally, improvements to **Pre-Surgical Rehabilitation Care,** was identified as a significant need. Multiple physiotherapists noted the current lack of available pre-surgical rehabilitation services in Saskatchewan, describing a major system gap. One participant stated. *“It’s a huge gap there because read all of the surgical reports, they say failed conservative measures. Well, nobody gets any pre-op physio. It just isn’t available.”* Patient perspectives mirrored this gap, echoing the lack of options for pre-surgical rehabilitation. HCPs specifically discussed the development of a structured pre-surgical rehabilitation program, where patients can receive pre-surgical education and rehabilitation from physiotherapists as a significant need. From an orthopedic surgeons point of view, the absence of such support during the waiting period was said to negatively impact patients’ mobility and QOL: *“My waitlist has over doubled in length. It’s over two years long now and people come in and their lives are destroyed because they’re waiting so long.”*

## Discussion

This research aimed to identify the unique needs, strengths, and preferences of First Nation and Métis Peoples in accessing and receiving care for TJR. We have identified a number of themes in patient participant interviews, including challenges faced whilst accessing care, support from family and home communities, pre-surgical experiences, impacts to QOL, and virtual care. HCPs identified barriers to accessing care, pre-surgical education, and future resource and implementation components. Saskatchewan has a substantial rural and remote population that faces inequities in accessing health care, which may impact pre-surgical TJR care.^10^ First Nation and Métis Peoples are disproportionally impacted by this, making up 17% of the population in Saskatchewan, and largely living in rural and remote areas.^9^ Access to pre-surgical care comes with numerous challenges, including travelling to receive care and financial expenses. Additionally, previous research suggests that healthcare access for Indigenous Peoples extends beyond geographic proximity to services, and is also shaped by social, cultural, and systemic factors.^38^ Higher rates of OA in Indigenous populations, compounded with inequities in accessing health care, contributes to increased disease burden and reduced QOL.^2,10^ During the study we worked in partnership with a Métis Elder and First Nation patient to help us identify experiences of Indigenous patients who were awaiting or had undergone hip and/or knee replacement surgery in Saskatchewan.

Patient participants overwhelmingly felt that a more comprehensive approach to pre-surgical education was a critical need, and should include the important concepts of language, culture, rehabilitation, and virtual care. Other studies have highlighted that language, cultural, and geographical barriers negatively affect care, recommending community-directed, patient-centered approaches to enhance equity and health outcomes.^39-41^ The major recommendations in this study to improve pre-surgical education for Indigenous Peoples in SK included care in community, culturally relevant care, pre-surgical rehabilitation, and virtual care. Reichert et al. (2023) also identified similar needs for culturally relevant care in community and possibilities of virtual care in their community needs assessment.^42^

Patient and HCP perspectives aligned in identifying travel, language, and financial barriers to accessing services, consistent with findings from previous research.^17,19,39,42^ A preference for care in the community was expressed, where patients could access regular appointments close to their support systems. Reichert et al. (2023) highlight the need for health care access and education in community, specifically identifying post-surgical physiotherapy as a critical need in rural and remote Indigenous communities in Saskatchewan.^42^ This aligns with current research, as Crockett et al. (2024) found that providing care closer to home facilitates healthcare access.^43^ However, it is important to note that care is most effective when developed in collaboration with Indigenous communities, with trusting relationships as the foundation.^41,44^

Culture was consistently discussed with patient participants, encompassing pre-surgical educational materials in First Nation and Métis languages, enhanced cultural awareness and understanding from HCP, and accessibility of cultural liaisons in health care centres. HCP acknowledged the relevance of cultural factors in patient care, but their current practices did not routinely integrate these considerations, identifying a potential gap between awareness and implementation. Consistent with our interpretation of culturally responsive care, participants in Loyola-Sanchez et al. (2020) emphasized the importance of healthcare providers understanding Indigenous cultures and histories to foster trust and meaningful communication.^41^ Longo et al. (2016), describes the benefit of psychological and emotional preparation pre-surgery, where education tailored to patients culture is most effective.^16^ Moreover, a lack of complete understanding of the TJR process resulted in feelings of fear and anxiety surrounding surgery. Existing research indicates that pre-surgical education reduces anxiety and improves patient satisfaction following hip or knee replacement surgery, suggesting more comprehensive education may further support patients in preparing for surgery.^16^

A lack of options to access pre-surgical rehabilitation was identified by both patients and HCP. Current clinical recommendations recommend exercise as a primary intervention for managing hip and knee OA.^14,15,45^ However, access to rehabilitation services, such as physiotherapy, is extremely limited in rural and remote areas, with only 2.8% of physiotherapists in Saskatchewan practicing in these regions as of 2023.^46^ HCPs reiterated that conservative measures such as exercise therapy are often unavailable to patients. Further, unless patient participants sought out physiotherapy at a private clinic independently, they had not accessed such support through public services. While pre-rehabilitation is generally perceived as beneficial by patients, current evidence is inconclusive regarding its effects on health outcomes such as function or pain.^47,48^ However, given their role as primary care providers, physiotherapists are well positioned to integrate OA education and mental health interventions into clinical practice, particularly for patients with chronic pain.^49^ Integrating mental health interventions may improve outcomes for patients with chronic conditions, including OA, by supporting coping strategies and enhancing overall QOL.^49^

Virtual care was identified by both patients and HCP as a strategy to overcome barriers to care, particularly for appointments that focus on patient education. These findings are consistent with the literature, which demonstrates that virtual care can reduce the burden of travel and facilitate access to specialized services.^17,18,50,51^ Irvine et al. (2022) reported a 91% satisfaction rate with virtual surgical consultations, with convenience being the most prominent qualitative finding, suggesting an effective alternative to in-person appointments.^52^ Family support was crucial for all patient participants, as they heavily relied on their support systems to cope with the recovery process. Virtual care can support family involvement by allowing patients to remain in their communities and include their support system in appointments. Family support at appointments can be impossible with in-person visits due to barriers such as travel challenges, lost wages from taking time off work, and childcare demands.

Recommendations from both patients and HCP to address knowledge gaps through improving equitable access to pre-surgical education included a more comprehensive and culturally responsive community-directed approach to pre-surgical education, as well as education and exercise materials in First Nation and Métis languages. Zhang et al. (2024) also identified the importance of communication and education in the management of chronic pain in a First Nation and Métis community.^39^ Recommendations also included continuation of community-directed co-development of virtual education programming through subsequent collaborative research, ongoing leadership by Indigenous patient participants, and inviting family members to ensure Indigenous voices are prioritized through subsequent stages of work.

To the best of our knowledge, this paper is the first of its kind to explore First Nation and Métis patient perspectives of pre-surgical TJR care and management of chronic arthritis. This project ensured uplifting of Indigenous knowledges and voices, including co-analysis with Indigenous patient partners, to ensure a patient-centered approach. The findings of this needs assessment led to the formation of a patient partner circle to co-develop a virtual pre-surgical education session to ensure culturally responsive information that will be relevant to community and their history as Indigenous Peoples in Saskatchewan. Moving forward, we will be including the option for Indigenous Peoples to indicate their specific background and language preference to ensure that recommendations and education is built in a distinction-based approach.^29^ As a result of the findings of this needs assessment, future recommendations also include co-development and evaluation of community-based service models that integrate cultural and community knowledges and support. Our team is currently investigating the use of virtual and hybrid models to support this area.

## Conclusion

First Nation and Métis participants who had received or were awaiting TJR in Saskatchewan identified the need for accessible, culturally responsive pre-surgical education and directed recommendations for future rehabilitation care. Interviews with First Nation and Métis patients and health care providers emphasized barriers they experience when accessing healthcare services in Saskatchewan. Language, financial constraints, and travel burdens were consistently raised by both patient and provider perspectives. Gaps in equitable access to healthcare were particularly evident between urban and rural patients, largely due to differences in proximity to and availability of pre-surgical care. Pre-surgical rehabilitation was widely inaccessible for both urban and rural patients, demonstrating a gap in exercise and OA education prior to TJR surgery. Patients noted profound levels of fear and anxiety before surgery due to knowledge gaps and experienced barriers. Overall, these findings emphasize the importance of comprehensive culturally responsive and community-directed education. Future research should explore expanding pre-surgical education in a manner directed by First Nation and Métis Peoples awaiting TJR surgeries in Saskatchewan.

## Limitations

While patient participants from various areas of Saskatchewan were interviewed, the results cannot be generalized to all First Nation and Métis patient experiences in the province. With seven participants, and six health care providers, the participant pool is likely not representative of the population. However, as noted, a number of procedures were undertaken to ensure information power and trustworthiness. These procedures included deep and rich stories shared by participants, member and peer checking, and the careful use of a codebook. Future research should include increased sample size in additional regions to gain greater understanding of Indigenous patient experiences. Although both Métis and First Nation participants were included, further description by nation was not reported, limiting generalizability to all Indigenous Peoples in Saskatchewan or Canada. The questionnaires used to collect demographic information were neither validated nor pilot tested. They were developed specifically for community-directed collaborative work with the participant group of interest in this study. While they were only used to collect descriptive information about participants, the absence of validation may have affected the accuracy and consistency of the demographic data collected.

## Supplemental Material

Supplemental Material - First Nation and Métis Needs Assessment for Pre-surgical Rehabilitation and Education of Hip and Knee Total Joint Replacements in Saskatchewan: Uplifting Indigenous Knowledges Supplemental material for First Nation and Métis Needs Assessment for Pre-Surgical Rehabilitation and Education of Hip and Knee Total Joint Replacements in Saskatchewan: Uplifting Indigenous Knowledges by Rebecca Sawatsky, Mervin Bouvier, Eric Honetschlager, Brenna Bath and Stacey Lovo in Health Services Insights.
