# Supplementary material for: First Nation and Métis Needs Assessment for Pre-Surgical Rehabilitation and Education of Hip and Knee Total Joint Replacements in Saskatchewan: Uplifting Indigenous Knowledges
Source: Health Serv Insights. 2026 Sep 9;19:11786329261488704. doi: 10.1177/11786329261488704 (PMC13558932; doi:10.1177/11786329261488704)
Supplement: Supplementary file 1 [file sj-pdf-1-his-10.1177_11786329261488704.pdf]

Appendices

Appendix 1 – First Nation and Métis Patient Interview Questions

Appendix I: Interview Guide for Pre-surgical Total Joint Replacement Patients

Preamble:

Hello, my name is X, and I am part of the research team looking at understanding total joint replacement pre-surgical experiences and perceived needs to improve pre-surgical care. Thank you for agreeing to talk to me about your experiences with pre-surgical assessment for total joint replacements.

As you know from the consent form we just reviewed together, the information you share today will help us to better understand: your personal experiences having a total joint replacement (both positive and negative), and your experience accessing pre-surgical assessment and education. If you are on a waitlist for a total joint replacement we want to hear about your perceived needs for pre-surgical assessment and if you anticipate any barriers to accessing pre-surgical care.

The information you share will help us to better understand what your pre-surgical assessment needs are and help to develop a culturally responsive way to provide pre-surgical assessment and education for Indigenous peoples in Saskatchewan. It may also be helpful to other people in a similar situation.

Your information will be combined with information shared by others from rural Indigenous communities who have also required a total joint replacement, health boards, indigenous community leaders and health care professionals with expertise in the area. We plan to share our findings with policy and decision makers. We hope the findings from this study will help improve accessibility and quality of pre-surgical care provided to Indigenous patients in Saskatchewan.

This interview/ discussion should take approximately 30 minutes to 1 hour.

As we reviewed in the consent form, I will be recording our conversation today and the recording will then be typed up into a written script for analysis after the research project is completed. Are you ok with proceeding with the interview at this time?

Do you have any questions before we get started?

If no...

Let's get started...I am turning on the recorder now.

A. Have you had a total joint replacement?

If Yes:

1. How many joint replacements have you had?

2. What joint replacement procedure(s) have you had?

3. Overall, how did you feel about your surgical experience?

a) Were you happy with how the surgery turned out? Why or why not?

4. How has having the joint replacement impacted your life?

a) Physical abilities?

b) Social Participation? (e.g. activities with family, friends, the community or work)

c) Emotionally?

d) Your ability to practice your culture?

5. Did you attend pre-surgical assessment/education sessions?

a) Which healthcare providers were involved in your care prior to surgery?

b) Were you provided with any treatment to help prepare you leading up to surgery? (e.g. exercise, medications)

6. Did the pre-surgical assessment help you feel more prepared for surgery?

a) Did you feel adequately educated on the surgical procedure before surgery? (e.g. process, recovery etc.)

b) Were you provided with educational materials and handouts to refer back to?

c) Please tell us anything additional that you would like to share about this experience.

If No:

1. How long have you been on a waitlist for a total joint replacement?

2. Have you been contacted for pre-surgical assessment appointments?

3. Which health care providers have been involved in your care so far?

4. Have you been educated on the surgical process?

5. Have you been provided with any treatment to prepare you for surgery? (e.g. exercise, medications)

6. Have you been provided with any equipment or resources prior to surgery? (e.g. walking aid, educational materials)

7. If your surgery were to happen today would you feel ready?

B. What types of supports/ services did you access prior to surgery? What has been helpful?

Prompts:

a) Health care services? (including physical therapy services…? medications?)

b) Community supports?

c) Others? (e.g. companion animals)

d) Local/traditional cultural practices?

C. How far would you typically have to drive to your nearest main center to access a pre-surgical appointment?

D. How far would you typically have to drive to access healthcare (if different)?

E. Can you tell me about any challenges you have had accessing care prior to or after surgery?

Prompts:

e) Travel?

f) Wait times?

g) Financial?

i. Need for accommodations? Food and/or per diem? Food reimbursement?

ii. Ability to pay for a companion to join you for your appointments?

h) Cultural?

i) Comfort interacting with health care professionals?

j) Other?

Additional prompt considering services responses above:

Which services were impacted by these challenges?

Is there anything additional you would like to share about this?

F. What types of other services and/or supports do you think would help/have helped to support you in understanding and feeling well prepared for surgery?

G. Can you tell me about things that have helped you receive pre-surgical care?

Prompts:

a. For example, something that helped remove financial barriers, or geographic/location barriers, wait times or cultural mismatch?

b. extra health insurance?

c. publicly funded service?

d. care in the community?

e. quick access to health care providers?

f. others?

H. What are things that are meaningful or important to you when it comes to improving access to pre-surgical care?

Prompts (Use terms in brackets):

a. affordability (costs, travel time, how you are able to pay for services)

b. availability and accessibility (location of services, hours of opening, appointment mechanisms, short wait times)

c. Appropriateness (Quality of care, trusting relationship, effectiveness of care)

d. Acceptability (professional values/beliefs are in line with your own, cultural awareness/understanding/connection, gender, languages that information is available in)

e. Approachability (honesty, respect)

I. If changes in health care services occurred in your community, do you have any suggestions on how we could measure if these changes were successful in improving access to and the quality of care?

Pause and use the following prompts if needed:

a. The experiences/stories of people who have experienced a total joint replacement?

b. Less Pain? Measuring pain levels?

c. Better quality of life? (Clarifying what types of activities you are now able to participate in, that you couldn't do before receiving appropriate health care...?)

d. Better movement/ mobility?

e. More able to participate in social/ community activities?

f. Less use of prescription medicines?

g. Less travel from the community?

h. Others?

J. Do you have any last ideas on how the pre-surgical assessment and education process could be improved to make the experience better for Indigenous patients?

K. How do you feel about technology and the idea of accessing healthcare virtually?

Prompts:

a. Do you believe virtual care would be accepted and viewed as beneficial in your community?

L. What is your preferred way to hear about the results of this study (e.g. mail, email)?

M. Is there anything else you would like to share with us about either your experiences with the pre-surgical process or accessing pre-surgical care?

Thanks so much for your time and your thoughts. Before we finish today, I would like to go back to the consent form briefly. Now that we have been through the interview and you know what you have shared with me, I just want to go back through the sections we checked off and see if you still consent in the same way as before we started. It’s perfectly OK to change your mind on any of this. [At this point confirm all the check box decisions with the participant]

Appendix 2 - Health Care Provider Interview Questions

Appendix K: Health Care Practitioner Interview Guide

Preamble:

Hello, my name is X, and I am part of the research team looking at understanding total joint replacement pre-surgical experiences and perceived needs to improve pre-surgical care for Indigenous patients. Thank you for agreeing to talk to me about your experiences with pre-surgical assessment for total joint replacements.

As you know from the consent form we just reviewed together, the information you share today will help us to better understand: your experience as a health care practitioner with pre-surgical assessment and education, your ideas for how the pre-surgical assessment can be improved, general feedback received from patients and any barriers you anticipate patients might have to accessing pre-surgical care.

The information you share will help us to better understand and develop a culturally responsive way to provide pre-surgical assessment and education for Indigenous peoples in Saskatchewan. It may also be helpful to other people in a similar situation.

Your information will be combined with information shared by rural Indigenous people who have had involvement with the pre-surgical assessment, health boards and indigenous community leaders. We plan to share our findings with policy and decision makers. We hope the findings from this study will help improve accessibility and quality of pre-surgical care provided to Indigenous patients in Saskatchewan.

We want to acknowledge the environment that we are in today with Covid-19. Your experience today may be very different from your initial experiences providing healthcare in this area. During our

interview, try to think of your long-term experiences and the big picture, and not just what is going on today. We know that the events this past year will have influenced your experience, and we would like to hear about your full experience, so hopefully our questions will allow you to share your full experience.

This interview/ discussion should take approximately 30 minutes to 1 hour.

As we reviewed in the consent form, I will be recording our conversation today and the recording will then be typed up into a written script for analysis after the research project is completed. Are you ok with proceeding with the interview at this time?

Do you have any questions before we get started?

If no...

Let's get started...I am turning on the recorder now.

1. What is your profession?

2. How many years of experience do you have with pre-surgical assessment and education, or management of total joint replacements?

3. How would you describe your role in the pre-surgical assessment of patients? What is involved in your assessment?

4. What do you educate patients on prior to surgery? Are any educational resources provided to them?

5. Do you prescribe any treatments for patients as part of the pre-surgical process?

6. Is access to pre-surgical assessment and education information equitable for Saskatchewan residents?

7. What types of supports/ services do you know of that patients can access prior to surgery? What have patients found helpful?

Prompts:

a. Health care services? (including physical therapy services…? medications?)

b. Community supports?

c. Others? (e.g. companion animals)

d. Local/traditional cultural practices?

8. Can you tell me about any challenges your patients have had accessing care prior to or after surgery?

Prompts:

a. Travel?

b. Wait times?

c. Financial?

i. Need for accommodations? Food and/or per diem? Food reimbursement?

ii. Ability to pay for a companion to join you for your appointments?

d. Cultural?

e. Language barrier?

f. Comfort interacting with health care professionals?

g. Other?

Additional prompt considering services responses above:

Which services were impacted by these challenges?

Is there anything additional you would like to share about this?

9. What types of other services and/or supports do you think would help to support your patients with the total joint replacement process?

10. Can you tell me about things that have helped your patients receive pre-surgical care?

Prompts:

a. For example, something that helped remove financial barriers, or geographic/location barriers, wait times or cultural mismatch?

b. extra health insurance?

c. publicly funded service?

d. care in the community?

e. quick access to health care providers?

f. others?

11. Do you have any ideas on how to make pre-surgical assessments and education sessions more accessible to your patients?

12. What are things that are meaningful or important to you when it comes to improving access to pre-surgical care?

Prompts (Use terms in brackets):

a. affordability (costs, travel time, how you are able to pay for services)

b. availability and accessibility (location of services, hours of opening, appointment mechanisms, short wait times)

c. Appropriateness (Quality of care, trusting relationship, effectiveness of care)

d. Acceptability (professional values/beliefs are in line with your own, cultural awareness/understanding/connection, gender, languages that information is available in)

e. Approachability (honesty, respect)

13. Over the past year, healthcare practices have been forced to change due to COVID-19. Have these changes impacted access for pre-surgical care for total joint replacements?

Prompts:

a) Have you been able to continue providing pre-surgical assessments?

b) Has the way in which your patients have received care changed? How?

c) What has been difficult about these changes?

d) What has been easier for you in accessing health care providers? (i.e. – appointments being held over the phone or videoconference)

14. Do you have any last ideas on how the pre-surgical assessment and education process could be improved to make the experience better for Indigenous patients in the future?

15. How do you feel about technology and the idea of practicing healthcare virtually?

Prompts:

b. Do you believe virtual care would be accepted and viewed as beneficial by your patients?

c. Do you believe virtual assessment and education would result in a similar quality of care as in face interactions? Do you believe your assessment can be as effective?

16. What is your preferred way to hear about the results of this study (e.g. mail, email)?

17. Is there anything else you would like to share with us about your experiences with the pre-surgical assessment, education and how to improve access to pre-surgical care?

a. (Once they stop talking) anything else we should know?

Thanks so much for your time and your thoughts. Before we finish today, I would like to go back to the consent form briefly. Now that we have been through the interview and you know what you have shared with me, I just want to go back through the sections we checked off and see if you still consent in

the same way as before we started. It’s perfectly OK to change your mind on any of this. [At this point confirm all the check box decisions with the participant]

Appendix 3 – First Nation and Métis Patient Demographic Questionnaire

Participant #: __________

Date: _________________

Patient Participant Demographic and General Health

Thank you for agreeing to participate in this study. Please answer the following questions to the best of your knowledge. To ensure confidentiality, please do not put your name on any of the following pages. If you have questions about the questionnaire, please speak to a member of the research team.

ABOUT YOU:

1. Age: ________

2. Which term(s) best describe your current gender identity? (Select the option(s) that apply to you).

Agender

Bigender/Multigender

Gender-fluid

Gender Queer

Man

Nonbinary

Questioning

Transgender

Two-Spirit

Woman

A gender not specified above. Specify: ____________

I prefer not to answer

3. Which sex was assigned to you at birth? (Select the option that applies to you).

Female

Male

I prefer not to answer

ABOUT YOUR HEALTH:

4. How long have you had problems with your joint pain (please indicate in months or years)?

5. Have you been diagnosed with osteoarthritis? (Select the option(s) that apply to you).

a. YES

b. Other diagnosis. Specify: _____________

6. Have you had a total joint replacement surgery?

IF YES:

a. What joint did you have replaced? (Select the option(s) that apply to you).

i. Hip

ii. Knee

iii. Other. Specify: _____________

IF NO:

b. Are you currently on the waitlist to have a total joint replacement surgery? (Select the option(s) that apply to you).

IF YES:

i. How long have you been on the waitlist to have total joint replacement surgery (please indicate in months or years)?

IF NO:

You do not fit the participant requirements for this project. Thank you for your time in filling out this survey.

c. What joint will you be having replaced? (Select the option(s) that apply to you).

i. Hip

ii. Knee

iii. Other. Specify: _____________

7. Does this problem limit your ability to participate in cultural practices? (Select the option that applies to you).

a. YES please describe:

b. NO

8. Are there any cultural or traditional methods you use for your hip and/or knee problems that you would like your health teams to understand?

Thank you for your time in completing this survey.

Appendix 4 – First Nation and Métis Patient Perspectives Themes:

Difficulty Accessing Care Theme:

Subtheme:	Patient Reponses:
Financial Strain of Healthcare Needs	P: “I think, making sure that they have their wheelchair assessment, or their different their walkers or whatever, making sure that they have access to that kind of information, so that way, you know, they're prepared. They're coming in for something they should they should prepare them and making sure that they have some kind of a liability or some kind of insurance to make sure that they're, they're covered, not to last minute. Oh, you're going to need this, you know. So, they should have it prepared.”
	P: “I was just spending money like crazy. Like I ran out of benefits, and it was, I was spending thousands on all these different things.”
Language: A Lot of People Don’t Understand	P: “With my mom, I always had to go with her because her English is so bad, having maybe an Indigenous interpreter”
Extensive Travel Requirements	P: “I was always able to get there. And my roommate's really good gives me a ride there. But I mean, it's tough. Gotta kind of plan it, because he's got so many chores to do and cows and stuff. But yeah, and of course, winter was really difficult because the roads you never knew, and you had to plan ahead and where we are. We if the grader hasn't come out and you're snowed in, there's not much you can do.”
	P: “Especially when they're on the reserve. Lots of them don't drive out there. I'm in the city, so it's different for me. I'm working and all the different things. So, it's easier for me. But for sure, I think of a lot of my family members that would have a hard time accessing proper health care without transportation and then the language barrier.”

Community and Family Support Theme:

Subtheme:	Patient Responses:
Community-Based Rehabilitation Needs	P: “We should have physiotherapists right in, right there, right away. But there’s not enough of them.”
	P: “It's not, it's not easy, you know, for a physiotherapist to carry a load as one, maybe a load as five or four or three, you know, it's good, but a load is one. It's very tough on a physio. You know, I could see the stress on the physiotherapist.”
Cultural Awareness and Understanding	P: “You know, you know, there's so many different areas and people, sometimes in a cultural setting or language. I think they don't understand

Subtheme:	Patient Responses:
	because they're they understand their ways of living, their culture, their language.”
	P: “I suppose if the person on the other side of the table was Indigenous, but that's harder too, because there's not a lot of Indigenous nurses and things any yet, but more.
Family Support: “I’m Glad I Had Somebody to Help Me”	P: “I think I have the proper care because I have a good wife.”

Pre-Surgical Experience Theme:

Subtheme:	Patient Response:
Appropriateness of Pre-Surgical Care	P: “My family doctor would refer me to the surgeon like pretty much every year, and they’d ask me what I was doing, and I’d tell them what I was doing, and they said well if you can still do that you should hold off on it. I say yeah but this really hurts when I do this.”
	P: “And I was like oh no, no, no, I don’t need this. And he was like “yeah, yeah, you do” … just me being in disbelief that that was real and that I really wasn’t like, I had almost gotten to a point where I was really believing that it was all in my head and that nothing was wrong with me, and I was just like, like seeking attention or something.”
Availability of Physiotherapy Services	P: “I was never sent to physio or anything about it.”
	P: I was seeing acupuncture therapists, a Bowen therapist, I did physio, I did massage, I did a naturopath. I tried literally everything that I thought was beneficial, but nothing really seemed to work.”
Variable Quality of Education Provided	P: “They explained it to me in a lot of different ways. You know, they but I didn't understand it. You know, because who understands about changing a hip and putting something in in your body, you know, because you're, you're you live with your own organs. You live with your own replacements. But a replacement, oh, man, it's hard to understand.”
	P: “I had some questions left unanswered, but I still went ahead and left anyway, and I thought, Well, might as well go and get it over with.”

Quality of Life Theme:

Subtheme:	Patient Response:
Cultural Participation	P: “Just not being able to join in on anything like any of our smudges, anything where we're sitting down around a fire, I can't sit down anymore.”
Experiencing Functional Decline	P: “Like I used to work on other stuff. Like, if I was go out and go out in a bush or something. Like, I used to live across the lake, and I'd walk on the lake, kind of go check the tip out stuff. I couldn't even walk, had to stop and sit in the snow for about a good half hour till my hip was good. And then I go again, do it again, stop again, about 10 times. Finally, I'm home, right?”
Emotional Burden of Chronic Arthritis	P: “It’s exhausting. Yeah, and being in pain, it’s exhausting, and it’s a lot”.
	P: “Yeah, it has impacted me in a lot of ways because of, uh, of that pain. You know, I couldn't, you know, sometimes it's just about angry. You know, you're angry that you can't do as much as you want. You know, because you can't go, you can't stand in one place for a while, you can't sit in one place for a while,

Subtheme:	Patient Response:
	because you're, you're in pain because you walked a little bit, or you lift something."
Post-Surgical Pain	P: "I try to avoid anything that I can't do if I can, like, push through work functions, if I have to, I push through, you know, stuff with my kids at their school, or whatever it might be. But lots of times I'll be like, I'm not coming. I'm busy that night, but I'm not I'm just going to sit at home, but it's because I can't, can't do it anymore."
	P: "After surgery, like I thought I was going to be like, no more pain. But it's not like that, so it's a lot of disappointment with that".
Patient-Centered Rehabilitation	P: "It would have helped with someone to assist me to make sure I was doing it the right way. But you couldn't have, you couldn't have someone in your home do it with you".
	P: "It was frustrating, because up in *northern town name redacted*, it was COVID … and the physio had quit at the hospital in *northern town name redacted*, and they didn't have a replacement. And my choice was to go to the city and get physio, which I couldn't because towing my mom back and forth and the fuel, it was just too hard to do."

296 Virtual Care Theme:

Theme: Virtual Care	Patient Response:
	"It would be very difficult, because we live in a remote setting … but the older people, they don't even, they don't even understand that part of it, you know, it'd be tough for them to go to the virtual part of it"
	"I only seen him with two, three minutes. He come in, he looked at me and I walked and then he said, We'll do surgery … just by looking at me and stuff like that, you know? And if you had virtual I probably would have been, but I had to go over there. You cost me money, and then with the storm, wintertime is hard."

297 Appendix 5 – Health Care Provider Perspectives Themes

298 Challenges Accessing Care Theme:

Subtheme:	Participant Responses:
Socioeconomic Barriers Faced by Patients	Orthopedic surgeon: "Socioeconomics play a huge role in someone's ability to maintain their health. And that's not only in pre-surgical care, but also in preventative care for arthritis."
	PT: "I would say getting equipment sometimes can be a bit of a challenge cause if they're status, they have to go through NIHB which is with funding, so sometimes that can be a bit of a challenge getting them a walker."
Concern About Impact of Language in Communication	Orthopedic surgeon: "Some people have significant language barriers and it's really difficult to optimize their care because it's hard to understand what's going on with them."
	PT: "I did have one patient who mostly spoke Cree. So, there was kind of a language barrier. We were able to communicate and didn't have too much trouble, but he'd always look towards his daughter who was translating for him. So, I mean,

Subtheme:	Participant Responses:
	probably things got misunderstood, I'm guessing."
Disproportionate Burden of Travel	PT: "I think that technology should be used a lot, lot more, especially in the far north where travel is such a patient burden and such a financial burden as well."
	PT: "Sometimes five minutes with an orthopedic surgeon when you're considering a 12 hour round trip on a paid taxi with hotels and meals just to have an x-ray and three- or five-minute conversation pre-op or post-op with an orthopedic surgeon."

Education Associated with Total Joint Replacements Theme:

Subtheme:	Participant Responses:
Comprehensive Pre-Surgical Education	PT: "People really know that the exercise portion of their post-op care is really, really important…but even telling them that pre-op, how important they are … getting them ready or maybe they get to the point where they can extend their OR date."
	Orthopedic surgeon: "I think the most important thing is understanding what is causing their dysfunction. So, educating them on the natural history of arthritis, what causes it, and why they feel the way that they do."
Accessible Educational Materials	Orthopedic surgeon: "There's something called a Cryro Cuff… but it's something you'd have to rent before, and it costs you a couple hundred dollars to do and so I have enough patients that can't afford – that really can't afford it. And so, when I'm building my website, I always have to think okay, do I actually put information on this on there because I don't [want] someone who can't really afford it to feel like they have to have it because it's on my website."
	PT: "I think another big thing with the surgery is if somebody has known somebody that has had it and the experiences that they have had always plays a really big role cause that's usually one of the number one thing … so those past experiences, who they've known who've had it kind of play a role in things."
Contemplating Virtual Care	PT: "With pre-op, generally we're delivery the same talk … we thought it was better actually having somebody talk rather than recording it and just having them go to a link because you still kind of get that face-to-face experience and they can still ask questions."
	Orthopedic surgeon: "90% of my job which is communicating the diagnosis and the pre-, inter-, post-op course and the risks and benefits, and all those types of things which is just me communicating my information to them, that's best done on a digital platform or virtual consult in my opinion."

Future Resource and Implementation Needs Theme:

Subtheme:	Participant Responses:
Care Available in the Community	PT: "I was chatting with a few of the other people, and we were kind of talking about it too and I think one thing is having our resources available in the communities that they're coming from would be helpful."
	PT: "I would say yeah, take me out there … that's gonna be the best healthcare that we can provide in my view."
Culturally Responsive Care	PT: "I think having culturally sensitive content or areas within the websites to be able to have that culturally specific content would be a huge thing. There's lots of things that can be done for sure."
Language Specific	PT: "That'd be helpful with a handful of our patients. Like I say, you have

Subtheme:	Participant Responses:
Resources	someone under 40, 50 years old, they can probably speak and read English relatively well, but beyond that, it's not always the case. So yeah, getting translated probably wouldn't be bad for some patients."
Pre-Surgical Rehabilitation Care	PT: "I think pre-op, having something available to help them either start on their exercises or give them some more education because they come in and they sign their consent and then they're not getting surgery for a year or two later. So, what can we do in between that waiting period?"

Supplementary File 1: COREQ checklist

Consolidated criteria for reporting qualitative studies (COREQ): 32-item checklist

Developed from:

Tong A, Sainsbury P, Craig J. Consolidated criteria for reporting qualitative research (COREQ): a 32-item checklist for interviews and focus groups. International Journal for Quality in Health Care. 2007. Volume 19, Number 6: pp. 349 – 357

Item No	Guide Questions/Description	Reported on Pag
Domain 1: Research team and reflexivity		
Personal Characteristics		
1. Interviewer/ facilitator	Which author/s conducted the interview or focus group?	8 ,9
2. Credentials	What were the researcher's credentials? E.g., PhD, MD	7
3. Occupation	What was their occupation at the time of the study?	7
4. Gender	Was the researcher male or female?	7, 8
5. Experience and training	What experience or training did the researcher have?	7
Relationship with participants		
6. Relationship established	Was a relationship established prior to study commencement?	8, 9
7. Participant knowledge of the interviewer	What did the participants know about the researcher? e.g. personal goals, reasons for doing the research?	8
8. Interviewer characteristics	What characteristics were reported about the interviewer/facilitator? e.g. Bias, assumptions, reasons and interests in the research topic	7
Domain 2: study design		
Theoretical framework		
9. Methodological orientation and Theory	What methodological orientation was stated to underpin the study? e.g. grounded theory, discourse analysis, ethnography, phenomenology, content analysis	9
Participant selection		
10. Sampling	How were participants selected? e.g., purposive, convenience, consecutive, snowball	8
11. Method of approach	How were participants approached? e.g., face-to-face, telephone, mail, email	8

Item No	Guide Questions/Description	Reported on Pag
Domain 1: Research team and reflexivity		
Personal Characteristics		
12. Sample size	How many participants were in the study?	12
13. Non-participation Setting	How many people refused to participate or dropped out? Reasons?	N/A
14. Setting of data collection	Where was the data collected? e.g., home, clinic, workplace	9
15. Presence of nonparticipants	Was anyone else present besides the participants and researchers?	N/A
16. Description of sample	What are the important characteristics of the sample? e.g. demographic data, date	11, 12
Data collection		
17. Interview guide	Were questions, prompts, and guides provided by the authors? Was it pilot tested?	9
18. Repeat interviews	Were repeat interviews carried out? If yes, how many?	N/A
19. Audio/visual recording	Did the research use audio or visual recording to collect the data?	9
20. Field notes	Were field notes made during and/or after the interview or focus group?	N/A
21. Duration	What was the duration of the interviews or focus group?	12, 13
22. Data saturation	Was data saturation discussed?	10
23. Transcripts returned	Were transcripts returned to participants for comment and/or correction?	7
Domain 3: analysis and findings		
Data analysis		
24. Number of data coders	How many data coders coded the data?	9, 10
25. Description of the coding tree	Did the authors provide a description of the coding tree?	N/A
26. Derivation of themes	Were themes identified in advance or derived from the data?	9, 10
27. Software	What software, if applicable, was used to manage the data?	9, 10
28. Participant checking	Did participants provide feedback on the findings?	10
Reporting		
29. Quotations presented	Were participant quotations presented to illustrate the themes/findings? Was each quotation identified? e.g., participant number	13-28
30. Data and findings consistent	Was there consistency between the data presented and the findings?	13-28
31. Clarity of major themes	Were major themes clearly presented in the findings?	13, 21
32. Clarity of minor themes	Is there a description of diverse cases or a discussion of minor themes?	13-28

307
